# Nitrogen and Phosphorus Dual-Doped Multilayer Graphene as Universal Anode for Full Carbon-Based Lithium and Potassium Ion Capacitors

**DOI:** 10.1007/s40820-019-0260-6

**Published:** 2019-04-03

**Authors:** Yuting Luan, Rong Hu, Yongzheng Fang, Kai Zhu, Kui Cheng, Jun Yan, Ke Ye, Guiling Wang, Dianxue Cao

**Affiliations:** Key Laboratory of Superlight Material and Surface Technology of Ministry of Education, College of Material Science and Chemical Engineering, Harbin Engineering University, Harbin, 150001 People’s Republic of China

**Keywords:** Arc discharge, Graphene, Heteroatom doping, Lithium/potassium ion battery, Lithium/potassium ion capacitor

## Abstract

Lithium/potassium ion capacitors (LICs/PICs) have been proposed to bridge the performance gap between high-energy batteries and high-power capacitors. However, their development is hindered by the choice, electrochemical performance, and preparation technique of the battery-type anode materials. Herein, a nitrogen and phosphorus dual-doped multilayer graphene (NPG) material is designed and synthesized through an arc discharge process, using low-cost graphite and solid nitrogen and phosphorus sources. When employed as the anode material, NPG exhibits high capacity, remarkable rate capability, and stable cycling performance in both lithium and potassium ion batteries. This excellent electrochemical performance is ascribed to the synergistic effect of nitrogen and phosphorus doping, which enhances the electrochemical conductivity, provides a higher number of ion storage sites, and leads to increased interlayer spacing. Full carbon-based NPG‖LiPF_6_‖active carbon (AC) LICs and NPG‖KPF_6_‖AC PICs are assembled and show excellent electrochemical performance, with competitive energy and power densities. This work provides a route for the large-scale production of dual-doped graphene as a universal anode material for high-performance alkali ion batteries and capacitors.

**Electronic supplementary material:**

The online version of this article (10.1007/s40820-019-0260-6) contains supplementary material, which is available to authorized users.

## Highlights

- Nitrogen and phosphorus dual-doped multilayer graphene (NPG) was prepared by arc discharge process.
- NPG exhibits good rate capability and stable cycling performance in both lithium and potassium ion batteries.
- Full carbon-based lithium/potassium ion capacitors are assembled and show excellent electrochemical performance.

**Electronic supplementary material:**

The online version of this article (10.1007/s40820-019-0260-6) contains supplementary material, which is available to authorized users.

## Graphical abstract

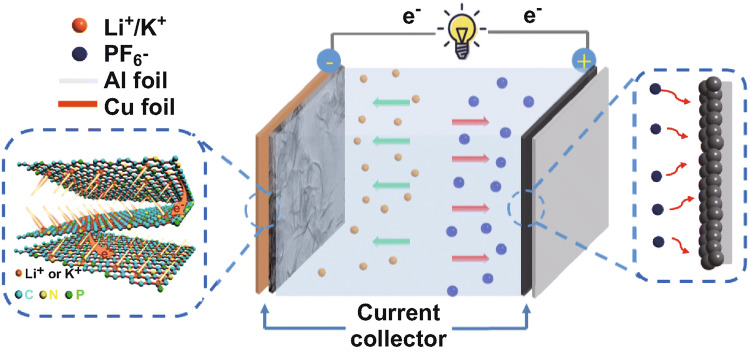

## Introduction

Lithium ion batteries (LIBs) are widely used in various types of portable electronic devices and vehicles, due to their high energy density and long cycle life. Nevertheless, the production costs of LIBs show an increasing trend, due to the limited availability of lithium sources [1]. Recently, potassium ion batteries (PIBs) have emerged as promising candidates for next-generation energy storage systems, owing to the larger potassium reserves in the earth’s crust and oceans and the low negative redox potential of the K^+^/K couple (− 2.931 V) [2]. Unfortunately, both LIBs and PIBs suffer from low power density and unsatisfactory cycling performance. To overcome these drawbacks, metal ion capacitors, a novel type of capacitor-battery hybrid energy storage systems, have been proposed to achieve high power and energy densities. Such devices are assembled by combining a high-energy battery-type anode and a high-power capacitor-type cathode [3]. During the charging process, cations are inserted in the anode by a Faradaic reaction and anions adsorb on the surface of the cathode by a non-Faradaic reaction; cations and anions return to the electrolyte in the discharging process. Considering the slow kinetics of the intercalation/extraction of metal ions, it is extremely important to explore battery-type anodes with high rate capability, to match that of the capacitive cathode.

Graphite is currently commercialized as an anode material for LIBs. However, its low theoretical specific capacity (372 mAh g^−1^) and poor rate performance cannot meet the increasingly high energy and power density demands of lithium ion capacitors (LICs) [4]. Graphene, a new type of carbon-based materials, displays remarkable rate capability and improved capacity for LIB applications, due to its large surface area and excellent electronic conductivity [5]. Recently, both theoretical and experimental studies showed that doping with heteroatoms is an efficient method to tailor the electronic structure and enhance the lithium ion storage properties [6, 7]. For instance, nitrogen-doped graphene sheets provide a higher reversible discharge capacity as LIB anode materials than pristine graphene [8]. The incorporation of nitrogen in graphene would further enhance the lithium ion storage and diffusion properties during cycling. Phosphorus, while being in the same VA group as nitrogen, has a higher electron-donating ability and a lower electronegativity. Graphene doped with a small concentration of phosphorus exhibits excellent electrochemical conductivity and electrochemical performance [9]. Moreover, Ju et al. reported that dual doping facilitates insertion and extraction of potassium ions [10]. Thus, dual-doped graphene would be a desirable anode material for LICs, as well as a potential anode material for potassium ion capacitors (PICs).

Heteroatom-doped graphene is usually synthesized through post-thermal treatment and direct synthesis. In the case of post-treatment methods, graphene or graphene oxide has to be obtained first, followed by treatment in NH_3_ or another atmosphere. The rigorous reaction conditions and toxic reactants greatly limit the practical applications of this approach [11, 12]. Due to the strong *π*–*π* interactions, irreversible stacking of graphene invariably occurs in the direct thermal annealing process [13, 14]. Therefore, the direct synthesis may represent a better choice. Currently, chemical vapor deposition is the most common direct synthesis method; unfortunately, high costs and low yields limit its application to produce heteroatom-doped graphene as an electrode material for large-scale energy storage systems. The production of doped graphene through a low-cost and high-yield method remains a challenging task.

Herein, a one-step arc discharge method is employed to obtain dual N, P heteroatom co-doped graphene (NPG) under He and H_2_ atmosphere, with (NH_4_)_3_PO_4_ as the source of nitrogen and phosphorus. The large-scale NPG nanosheets are composed of 2–6 graphene layers, with P and N concentrations of about 1.3 and 3.2 at.%, respectively. Owing to its remarkable structure and uniform heteroatom doping, NPG displays an extraordinary electrochemical performance as an anode material for LIBs. The enhanced lithium storage properties of NPG are attributed to the synergistic effect of the nitrogen and phosphorus dopants. When employed as anode for PIBs, NPG also shows high capacity, good rate capability, and stable cycling performance. Moreover, full carbon-based LICs and PICs are assembled using the as-prepared NPG as anode and active carbon (AC) as cathode. Both LICs and PICs exhibit a high voltage window, high energy density, and remarkable power density, demonstrating their potential applications. This work introduces a facile arc discharge approach to prepare graphene doped with different heteroatoms, with promising potential as an anode material. In addition, the present work may provide a new route to design high-performance electrode materials for not only Li/K ion batteries or capacitors, but also other energy storage systems.

## Experimental

### Preparation of Nitrogen- and Phosphorus-Doped Graphene Sheets

The NPG material was prepared by a one-step arc discharge method. In a typical procedure, graphite powders were first compressed into a rod, and an opening was drilled in the center of the rod. (NH_4_)_3_PO_4_ powders were then loaded into the cavity of the graphite rod as the raw materials, and the resulting rod was used as the cathode. At the same time, a pure graphite rod served as the anode. The cathode and anode were placed in a vacuum chamber pumped to below 1 Pa. The arc discharge process was carried out in a water-cooled stainless-steel chamber under a steady current of 120 A and a voltage of 15 V. Hydrogen and helium were introduced into the pre-vacuumed chamber as working atmospheres at a volume ratio of 1:3; both gases had 99.99% purity. NPG was obtained after cooling the cavity to room temperature. For comparison, N-doped graphene (NG) was obtained by introducing NH_3_ into the reactant gas in the absence of (NH_4_)_3_PO_4_, while P-doped graphene (PG) was obtained by introducing the same number of moles of phosphate instead of (NH_4_)_3_PO_4_. Pure graphene was obtained under the same conditions, without (NH_4_)_3_PO_4_. Graphene sheets were exfoliated by arc discharge in a buffer gas mixture of H_2_ (10 kPa) and He (90 kPa).

### Structure Characterization

X-ray diffraction (XRD) patterns were obtained with a Siemens D500 diffractometer, to investigate the crystal structure of the samples. The surface morphologies and detailed microstructures were analyzed by scanning electron microscopy (SEM, Nova NanoSEM 230) and transmission electron microscopy (TEM, FEI Tecnai G2 F20). The Raman spectra were obtained using a LabRAM HR800 confocal microscopic spectrometer with a laser excitation wavelength of 532 nm. The surface elemental composition and bonding configuration were determined by X-ray photoelectron spectroscopy (XPS, K-Alpha 1063, Thermo Scientific™). Thermogravimetric analysis (TGA) was carried out with a Netzsch STA 449C thermal analyzer.

### Electrochemical Measurements

The electrochemical performances were tested using CR2032 coin-type cells. The electrode materials were prepared by mixing the active material, Super P conductive additive, and the polyvinylidene fluoride binder at 8:1:1 weight ratio in the *N*-methylpyrrolidinone solution. The mixtures were mixed evenly, coated onto copper foil substrates and dried at 110 °C for 24 h in a vacuum oven; the cells were then assembled in an argon-filled glove box. The mass loading of the active material was about 1 mg cm^−2^. The electrolytes used for LIBs and PIBs were 1 M LiPF_6_ and 1 M KPF_6_, respectively, in ethylene carbonate/diethyl carbonate (1:1, *v*/*v*). Polypropylene film and lithium foil were used as the separator and counter electrode, respectively, for LIBs. A glass microfiber filter film and potassium foil were used as the separator and counter electrode, respectively, for PIBs. Charge/discharge tests were performed in the 0.01–3.0 V voltage window at various current densities, using a Neware battery tester. Cyclic voltammetry (CV) and electrochemical impedance spectroscopy (EIS) measurements of the electrodes were carried out on a Bio-Liogic VMP3 workstation. The CV measurements were conducted with a sweep rate of 0.1 mV s^−1^ in the range of 0.01–3.0 V. The frequency range of the EIS tests was set to 0.01–100 kHz, with a 5 mV amplitude.

Before assembling the LICs and PICs, the NPG half-cells were tested by lithiation at 0.02 V followed by delithiation at 3.0 V and 100 mA g^−1^ over five cycles, with a final lithiation at 1.0 V. LICs and PICs were then assembled with AC as the cathode and activated NPG as the anode in cathode/anode mass ratios of 1:1, 2:1, 4:1, 6:1, and 8:1.

The specific capacities (*C*, mAh g^−1^) of both half and full cells were derived from galvanostatic charge/discharge (GCD) curves according to Eq. 1:

1 $$C = I \times t/m$$

in which *I* is the discharge current, *t* is the discharge time, and *m* is the mass of AC. The energy density (*E*, Wh kg^−1^) and power density (*P*, W kg^−1^) of the LIC and PIC systems were calculated from the GCD curves according to Eqs. 2 and 3 [36]:

2 $$E = \frac{I\smallint V\left( t \right)d\left( t \right)}{m}$$

3 $$P = E/t$$

where *V*(*t*) is the potential of the device and *m* is the total mass of NPG and AC.

## Results and Discussion

**Fig. 1:**
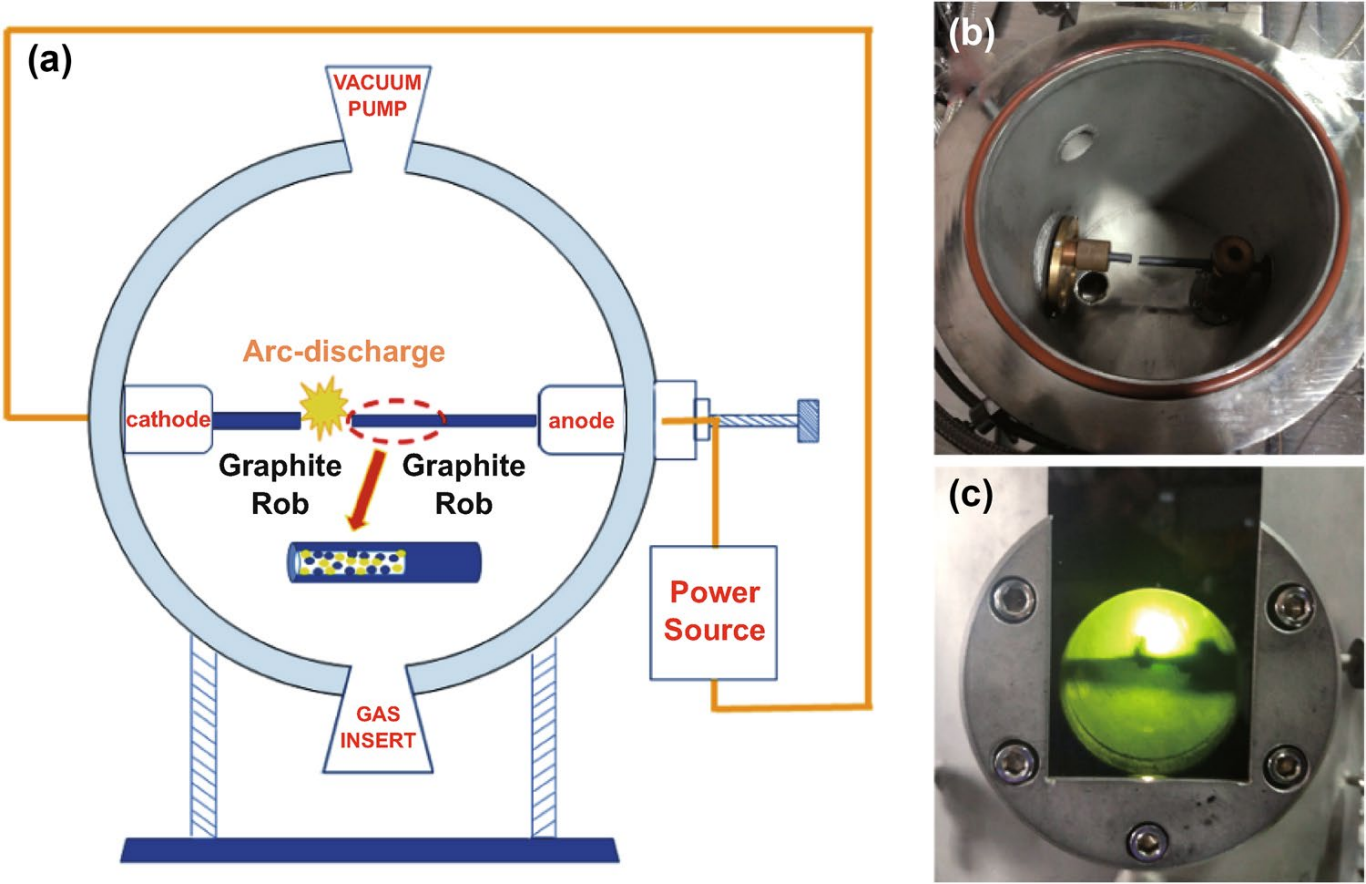
**a** Schematic diagram of the arc discharge process and **b** digital image of the arc discharge system; **c** typical photograph of the arc plasma

The synthetic strategy for the preparation of NPG is schematically illustrated in Fig. 1. In a typical procedure, NPG was prepared via the one-step arc discharge method (Fig. 1a). Digital images of the arc discharge system and a typical photograph of the arc plasma are shown in Fig. 1b, c. The graphite powder was compressed into a rod, and an opening was drilled in the center of the rod. Then, the pore was filled with solid (NH_4_)_3_PO_4_, which was used as both nitrogen and phosphorus source. Due to the enormous cooling gradient under the arc discharge process, the nitrogen and phosphorus atoms could be isolated from (NH_4_)_3_PO_4_ and rapidly doped into the graphene structure. In the case of the NG and PG samples, nitrogen and phosphorus atoms were generated from NH_3_ and pure red phosphorus, respectively. Defect sites would be generated during the doping process, via the adsorption of dissociated oxygen atoms on the surface of graphene.

**Fig. 2:**
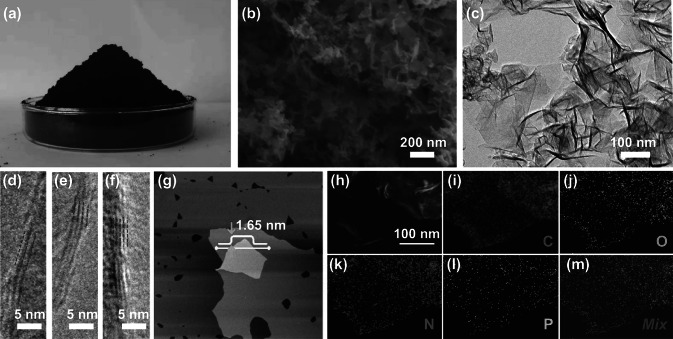
**a** NPG powder on a culture dish. **b** SEM and **c** TEM images of NPG nanosheets. High-resolution TEM images of NPG nanosheets with **d** two, **e** three, and **f** five graphene layers. **g** AFM image and height profile of NPG nanosheets. **h** Corresponding mapping images and that of **i** carbon, **j** oxygen, **k** nitrogen, and **l** phosphorus elements along with their superimposition (**m**)

The arc discharge exfoliation protocol is a fast and straightforward route to achieve mass production of doped graphene. The output depends on the size of the graphite rod and the reaction time. In this work, 1.0 g NPG (Fig. 2a) was prepared in 15 min, which is much shorter than that corresponding to the time-consuming Hummers’ method. Moreover, the yield of NPG was also larger than that of chemical vapor deposition at high temperature. The surface morphologies and detailed microstructures of the samples were analyzed by SEM and TEM. As shown in Fig. 2b, c, the as-prepared ultrathin NPG nanosheets appear as naturally crumpled and curly lamellar petals. Large-area multilayer graphene sheets with sizes of 100–500 nm are observed in Fig. 2c. The absence of amorphous carbon and other structures indicates the high purity of the graphene sheets that can be successfully produced by the arc discharge method. In addition, the wrinkled surface of NPG results in different levels of transparency. Hence, the layers can be distinguished through the folded edge, as shown in Fig. 2d–f. Most graphene sheets consist of six or less layers. Moreover, the atomic force microscopy (AFM) measurements in Fig. 2g highlight a uniform thickness of 1.65 nm, corresponding to approximately four layers, for typical NPG nanosheets [15, 16]. The homogeneous incorporation of nitrogen and phosphorus in NPG was highlighted by the elemental mapping images in Fig. 2h–m, which suggest uniform doping. The presence of oxygen is mainly due to the adsorbed oxygen species and surface oxidation. In addition, NPG shows a larger surface area than that of graphene, further indicating that the introduction of heteroatoms in graphene by the arc discharge method can effectively avoid the aggregation of graphene nanosheets (Fig. S1 and Table S1).

**Fig. 3:**
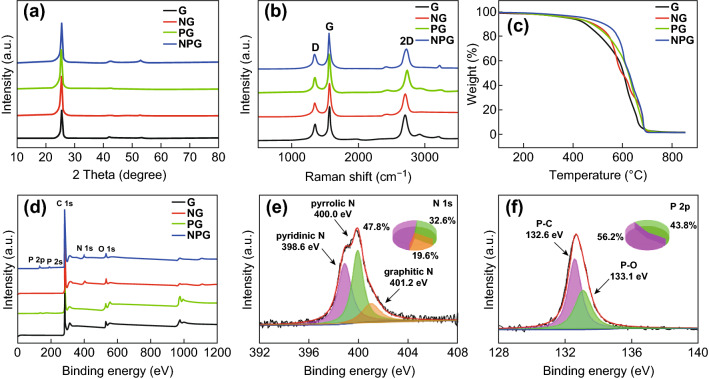
**a** XRD patterns, **b** Raman spectra, **c** TGA curves, as well as **d** full and high-resolution **e** N 1*s* and **f** P 2*p* XPS spectra of the NPG sample

The XRD analysis was used to characterize the crystal structure of NPG, NG, PG, and graphene sheets, as shown in Fig. 3a. The sharp and intense peak located at 2*θ* = 26.1° indicates a highly organized crystal structure, such as that of standard graphene [17]. Moreover, the peaks of NG, PG, and NPG are shifted to low angles, suggesting that doping with heteroatoms leads to an increase in the interlayer spacing (Fig. S2). Figure 3b shows the Raman spectra of the samples. The three dominant peaks located at ~ 1350, 1560, and 2600 cm^−1^ can be assigned to the D, G, and 2D bands, respectively. The G band is ascribed to the zone center *E*_2*g*_ mode related to phonon vibrations in *sp*^2^ carbon materials [18]. The D band is related to defects and disordered carbon structures. The ratio of the intensities of the G and D bands (*I*_G_/*I*_D_) corresponds to the amount of defects in graphene [19]. The integrated *I*_G_/*I*_D_ intensity ratios of the G, NG, PG, and NPG products are 2.26, 2.32, 2.38, and 2.31, respectively. The defects present in pristine graphene can be doped or replaced with nitrogen and phosphorus, resulting in little changes in the *I*_G_/*I*_D_ ratio. Moreover, the *I*_G_/*I*_2D_ ratio can be used to determine the number of graphene layers, with smaller *I*_G_/*I*_2D_ values denoting fewer layers [20]. As shown in Fig. 3b, the integrated *I*_G_/*I*_2D_ intensity ratios of the four products are calculated to be ~ 0.75, suggesting that the obtained graphene sheets are mainly composed of 2–6 layers [21], which is consistent with the results of the TEM analysis. Moreover, the TGA curves of the G, NG, PG, and NPG are shown in Fig. 3c. When the weight loss reaches approximately 20%, the mass of the samples declines rapidly, indicating the beginning of the decomposition. All samples exhibit a single mass loss step, at variance with graphene produced by chemical methods, which shows an additional step corresponding to the removal of oxygen functional groups. This result suggests that the as-prepared graphene samples possess high purity and crystallinity. XPS measurements were carried out to further investigate the elemental compositions and bonding modes of nitrogen and phosphorus in NPG, NG, and PG. The XPS survey spectrum of NPG is shown in Fig. 3d; the four characteristic peaks observed at ~ 285, 532, 131, and 401 eV correspond to the C 1*s*, O 1*s*, P 2*p*, and N 1*s* signals, respectively, indicating the successful P and N doping in graphene. The analysis shows that the amounts of nitrogen and phosphorus doped into graphene are 3.2 and 1.3 at.%, respectively. High-resolution C 1*s*, N 1*s*, and P 2*p* XPS spectra of NPG were also collected to gain further insight into the nitrogen and phosphorus doping. The peaks located at 284.6, 285.8, and 287.3 eV in Fig. S3 can be attributed to C–C, C–P, and C=N groups [22, 23], respectively, confirming the successful doping of nitrogen and phosphorus. The peaks located at 398.6, 400.0, and 401.2 eV in Fig. 3e can be assigned to pyridinic, pyrrolic, and graphitic N bonds, respectively, whose calculated percentages are 47.8%, 32.6%, and 19.6%, respectively. Pyridinic and pyrrolic N species are more active than graphitic N ones and can enhance the electronic conductivity and lithium kinetics. Furthermore, the peaks located at 132.6 and 133.1 eV in Fig. 3f can be assigned to P–C and P–O bonds, respectively [24]. The presence of P–O bonds favors the adsorption and accelerates the transport of lithium ions [25]. For comparison, the high-resolution C 1*s*, N 1*s*, and P 2*p* XPS spectra of NG and PG are shown in Figs. S4, S5, respectively. The spectra of NG, PG, and G are identical in all respects, except for the presence of nitrogen and phosphorus in NG and PG. The amount of nitrogen and phosphorus doped into NG and PG, as estimated by the elemental analysis of the XPS data, are 3.31 and 1.18 at.%, respectively.

**Fig. 4:**
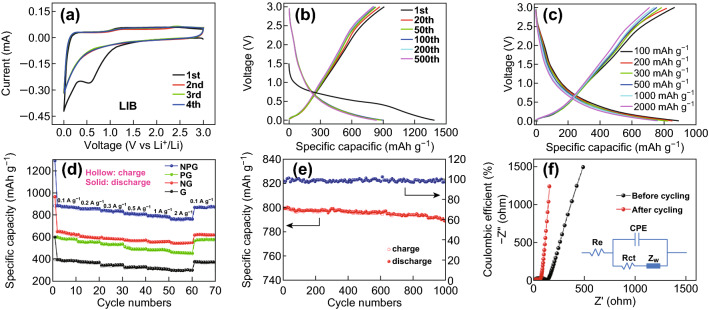
**a** CV curves of the NPG sample at a scanning rate of 0.1 mV s^−1^. **b** Galvanostatic curves of NPG at a current density of 100 mA g^−1^. **c** Typical galvanostatic curves of the NPG electrode at different current densities. **d** Rate capabilities of G, NG, PG, and NPG electrodes at different current densities. **e** Cycling stability of the NPG electrode at the current density of 1000 mA g^−1^. **f** Nyquist plots of NPG

To investigate the electrochemical performance, CV tests were carried out at a scan rate of 0.1 mV s^−1^, as shown in Fig. 4a. In the first cycle, the CV curve exhibits a large cathodic peak at about 0.55 V, which disappears during the following cycles. The irreversible peak can be attributed to the formation of a solid electrolyte interphase (SEI) layer and to the irreversible lithium ion storage on defect/edge sites of NPG during the initial cycle. The exact match between the areas of the second to the fourth cycle indicates a highly reversible lithium ion insertion/deintercalation process and a good capacity retention of NPG during the following cycles [26, 27]. Figure 4b shows the galvanostatic discharge–charge profiles of NPG at the current density of 100 mA g^−1^ in the voltage range from 0.01 to 3 V, measured in the 20th, 50th, 100th, 200th, and 500th cycle. In the first cycle, the NPG electrode delivers a lithiation capacity of 1383 mAh g^−1^ and a charge capacity 859 mAh g^−1^ at a current density of 100 mA g^−1^. The irreversible capacity is due to the SEI formation and to the irreversible lithium storage, in agreement with the CV results. For comparison, NG shows initial discharge and charge capacities of 982 and 636 mAh g^−1^, respectively, while the values obtained for PG are 913 and 576 mAh g^−1^, respectively (Fig. S6), which are much lower than those of NPG. This suggests that co-doping with heteroatoms could provide a higher number of lithium ion storage sites and an improved capacity. In addition, the profiles largely maintain their shape with increasing number of discharge–charge cycles, demonstrating a good structural stability. Figure 4c displays the lithiation/delithiation curves of NPG under different current densities, ranging from 100 to 2000 mA g^−1^. A reversible discharge capacity of 758 mAh g^−1^ is obtained even at a high current density of 2000 mA g^−1^, denoting a high rate performance. A potential plateau is observed at under 0.5 V, which can enhance the working voltage of the full cell. Figure 4d displays the rate performances of G, NG, PG, and NPG at various current densities. NPG shows reversible capacities of 889 and 758 mAh g^−1^ at current densities of 100 and 2000 mA g^−1^, respectively, which is higher than those of undoped G, NG, and PG, respectively. Owing to the similar morphology and structure of NPG, G, NG, and PG, the enhancement of the reversible electrochemical performance observed for NPG should be attributed to the N and P doping. Furthermore, all samples exhibit a small capacity loss at each current density (100, 200, 300, 500, 1000, and 2000 mA g^−1^) and a nearly 100% recovery of discharge and charge capacities when the rate is restored to 100 mA g^−1^, demonstrating a remarkable rate capability. Moreover, the stable capacity of NPG obtained at each current rate is also higher than that of the G, NG, and PG samples. The enhanced capacity of NPG is ascribed to the synergistic effect of phosphorus and nitrogen co-doping in graphene. The capacity enhancement is closely related to the doping content of nitrogen or phosphorus. As shown in Fig. S7, the NPG sample displays not only an enhanced capacity upon doping with nitrogen and phosphorus, but also a further increase in capacity due to the synergistic effect of N and P co-doping. Figure 4e shows the cycling performance and corresponding coulombic efficiency of NPG at a current density of 1000 mA g^−1^. A reversible capacity of 798 mAh g^−1^ is achieved in the initial cycles. After 1000 cycles, NPG retains a capacity of 787 mAh g^−1^, corresponding to a retention of 98.6% and a fading of 0.0014% per cycle. Moreover, NPG displays a stable coulombic efficiency of ~ 100% during the whole cycling process, with the exception of the initial cycles. For comparison, Fig. S6 shows that the discharge capacities of G, PG, and NG are about 280, 570, and 627 mAh g^−1^, respectively, at the same current density, confirming that heteroatom doping can improve the capacity of graphene. The AC impedance of NPG before and after cycling is shown in Fig. 4f. After cycling, the charge-transfer resistance of NPG decreases from 140.7 to 58.3 Ω (Table S2), due to the full penetration of the electrolyte and the activation of active materials, which promote fast lithium ion diffusion and efficient charge transfer at the electrolyte/electrode interface. Moreover, the comparison of the electrochemical performance of various N-doped, P-doped, and N, P co-doped carbon anodes in Table S3 highlights the competitive electrochemical performance of the as-prepared NPG.

**Fig. 5:**
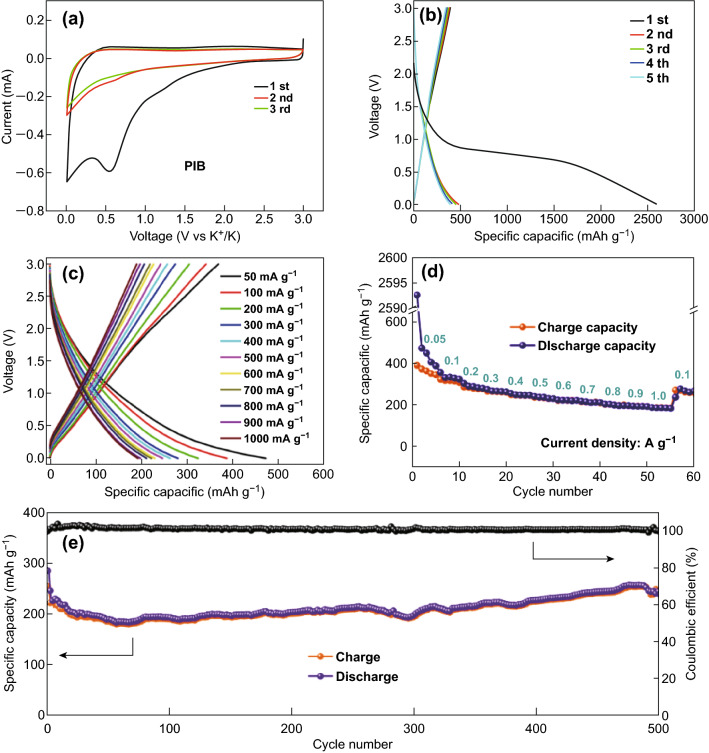
**a** CV curves of NPG at a scanning rate of 0.2 mV s^−1^. **b** Charge–discharge voltage profiles at a current density of 50 mA g^−1^. **c** Typical galvanostatic lithiation/delithiation curves and **d** rate capabilities of NPG electrode at different current densities. **e** Cycling stability and coulombic efficiency of NPG electrode at a current density of 500 mA g^−1^

Potassium ion batteries have attracted considerable attention, due to their low cost and potential application in large-scale energy storage systems. Thus, the potassium storage behavior of NPG was also investigated by CV. As shown in Fig. 5a, NPG shows similar CV curves in the first three cycles, but also a very large irreversible peak in the first cycle. Due to the similar potentials of the K^+^/K and Li^+^/Li couples, the irreversible peak is located at 0.6 V. In addition, the CV curves retain a similar shape in the second and third cycle, indicating a highly reversible potassium ion storage behavior in the NPG sample. Galvanostatic charge–discharge tests were carried out to further investigate the electrochemical performance of NPG in PIBs. As shown in Fig. 5b, the initial discharge and charge capacities are 2592 and 387 mAh g^−1^, respectively. The large irreversible capacity is due to the formation of the SEI layer and the irreversible potassium ion storage on defect and edge sites of the NPG. Considering that the atomic radius of potassium ions (1.38 Å) is larger than that of lithium ions (0.76 Å), NPG provides a lower number of ion storage sites, leading to more potassium ions irreversibly binding to the defect and edge sites of NPG; thus, NPG shows a lower capacity and initial coulombic efficiency. A coulombic efficiency of 98% is obtained for the NPG electrode during cycling, except for the first cycle, indicating the high reversibility of the intercalation/extraction process of potassium ions. Moreover, the rate capability of the NPG sample is shown in Fig. 5d. The capacities of the NPG electrode at current densities of 50, 100, 200, 500, and 1000 mA g^−1^ are 387, 324, 280, 247, and 194 mAh g^−1^, respectively. When the current density is switched back to 200 mA g^−1^, the discharge capacity returns to 270 mAh g^−1^, highlighting an outstanding rate capability (Fig. S8). At the same time, the discharge–charge profiles maintain similar shapes at various current densities, confirming the good rate performance and structural stability. The NPG electrode also exhibits a remarkable cycling stability, as shown in Fig. 5e. A reversible capacity of 232 mAh g^−1^ is achieved at the current density of 500 mA g^−1^. The capacity shows a slow decrease in the first 50 cycles, followed by a gradual increase due to the activation process during cycling. A discharge capacity of 242 mAh g^−1^ is obtained after 500 cycles. NPG also shows a stable coulombic efficiency of ~ 100%, except for the initial cycles. This indicates that potassium ions could be reversibly stored in NPG, which thus represents a promising anode material for PIBs [28, 29].

The high reversible capacity, rate performance, and cycling ability of NPG as the anode material for both LIBs and PIBs can be attributed to the intrinsic properties of graphene and the synergistic effect of N and P doping. First, the arc discharge method could produce high-quality and few-layer graphene nanosheets. The small thickness could provide shorter diffusion paths for lithium/potassium ions, and the large surface area enhances the contact between active materials and electrolyte. Second, doping with nitrogen atoms introduces active sites in graphene, providing additional storage sites for lithium/potassium ions [30, 31]. At the same time, nitrogen doping would enhance the electronic conductivity, leading to a high rate capability. Third, doping phosphorus atoms with large atomic radius can suppress the agglomeration of graphene [16, 32, 33]. The presence of phosphorus leads to the adsorption of lithium/potassium ions [25]. Therefore, NPG displays outstanding electrochemical performance.

**Fig. 6:**
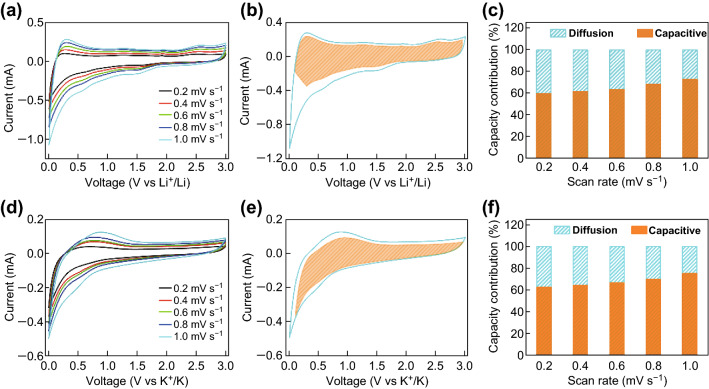
**a**, **d** CV curves of NPG at different scan rates. **b**, **e** CV profiles showing the capacitive contribution (filled part) of NPG at a scan rate of 1 mV s^−1^. **c**, **f** Diagram of the capacitive contribution to the total capacity at different scan rates. The (**a**–**c**) and (**d**–**f**) panels correspond to LIBs and PIBs, respectively

**Fig. 7:**
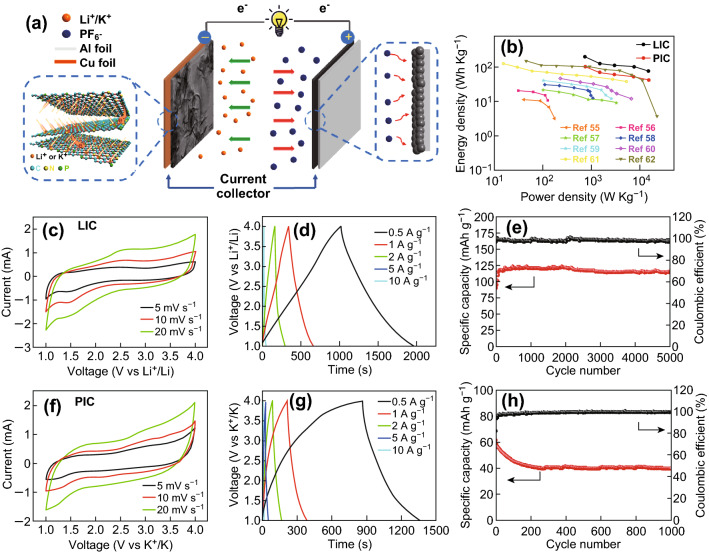
**a** Schematic illustration of the working mechanism. **b** Ragone plots compared with literature values at the optimal cathode/anode mass ratio of 6:1. **c**, **f** CV curves at scan rates of 5, 10, and 20 mV s^−1^. **d**, **g** Galvanostatic charge–discharge curves at current densities of 0.5–10 A g^−1^. **e**, **h** Cycling stability over 5000 and 1000 cycles at a current density of 1 A g^−1^

The kinetics of lithium ion storage in the NPG material was investigated by examining the CV profiles at different scan rates (*v*) (Fig. 6a). The current value (*i*) at a given potential (*V*) can be determined from the diffusion-controlled and surface capacitive contributions (*k*_2_*v*^1/2^ and *k*_1_*v*, respectively) on the basis of the following equation: *i*(*V*) = *k*_1_*v* + *k*_2_*v*^1/2^ [26–28]. The fractions of current originating from the capacitance and diffusion contributions could be distinguished by defining the *k*_1_ and *k*_2_ constants. The typical profile in Fig. 6b reveals that the capacitive contribution dominates the total capacity. Furthermore, the fraction of capacitive capacity progressively increases from 59.7% at 0.2 mV s^−1^ to a maximum value of 72.8% at 1 mV s^−1^ (Fig. 6c). Similarly, the capacitive capacity fraction increases with increasing scanning rate also in the case of potassium ion storage, and a maximum value of 75.1% is reached at a scan rate of 1.0 mV s^−1^ (Fig. 6f). This feature is highly beneficial for the fast transport of Li^+^ and K^+^ ions, leading to a highly reversible capacity and long cycle life. To investigate the potential applications of NPG, full carbon-based LICs and PICs were designed and assembled using NPG as anode and AC as cathode, as shown in Fig. 7a. During the charge process, lithium or potassium ions intercalate into the NPG anode and PF_6_^−^ ions are accumulated on the AC surface. A high voltage window of 1–4 V is achieved for the LICs, as shown in Fig. 7c. The high working voltage of the LICs can provide a high energy density. The CV profiles of the LICs display a quasi-rectangular shape at different scan rates, suggesting a high rate capability. As shown in Fig. S9, the CV profiles of AC exhibit a typical rectangular shape in the voltage range of 3–4 V, denoting its ability to absorb PF_6_^−^. According to the CV curves in Fig. S10, the optimal mass ratio of cathode and anode is 1:6. The CV profiles of LICs also display a quasi-rectangular shape at different scan rates, indicating their good rate capability. Figure 7d shows the charge–discharge curves at different current densities. The CV profiles of the LICs reveal capacities of 156, 98, 88, 83, and 61 mAh g^−1^ at densities of 0.5, 1, 2, 5, and 10 A g^−1^, respectively. The LICs show desirable symmetric characteristics with low *IR* drops. In addition, the LICs exhibit a good cycling performance at the current density of 1 A g^−1^. A high capacity of 117.8 mAh g^−1^ is achieved after 5000 cycles; the increase in capacity is mainly due to the activation of the cell. In addition, the PICs also exhibit good electrochemical performance. As shown in Fig. 7f, their CV curves display a quasi-rectangular shape, due to the similar chemical properties of potassium and lithium. The low *iR* drop in Fig. 7g demonstrates the small electrochemical resistance of the PIBs, which leads to a good rate capability. Due to the larger atomic weight of potassium ions, the PICs display a lower capacity than the LICs. In particular, capacities of 104.4, 68, 60.8, 52.5, and 41.6 mAh g^−1^ are observed at current densities of 0.5, 1, 2, 5, and 10 A g^−1^, respectively, and a capacity of 44.2 mAh g^−1^ is retained after 1000 cycles. The Ragone plots of LICs and PICs are shown in Fig. 7b. The NPG‖LiPF_6_‖AC LICs exhibit a maximum energy density of 195 Wh kg^−1^ with a power density of 746.2 W kg^−1^ at a current density of 0.5 A g^−1^, along with a maximum power density of 14,983.7 W kg^−1^ with an energy density of 77 Wh kg^−1^ at a current density of 10 A g^−1^. The NPG‖KPF_6_‖AC PICs show a maximum energy density of 104.4 Wh kg^−1^ with a power density of 760.6 W kg^−1^ at the current density of 0.5 A g^−1^, as well as a maximum power density of 14,976 W kg^−1^ with an energy density of 41.6 Wh kg^−1^ at a current density of 10 A g^−1^. Overall, the full carbon-based LICs and PICs display energy and power densities competitive with those of graphene–nickel cobaltite nanocomposite//AC [34], AC//sodium titanate nanotubes [35], graphite//AC [36], TiO_2_/reduced graphene oxide//AC [37], peanut shell derived carbon//Na_2_Ti_3_O_7_ [38], LiNi_0.5_Co_0.2_Mn_0.3_O_2_//AC [39], nitrogen-enriched carbon nanospheres/graphene//prelithiated microcrystalline graphite [40], and TiO_2_//CNT-AC [41].

## Conclusions

An anode material based on nitrogen and phosphorus dual-doped graphene has been successfully designed and synthesized via a one-step arc discharge method. NPG can be employed as a universal anode material for lithium ion and potassium ion batteries. As anode for LIBs, NPG displays a high specific capacity of 889 mAh g^−1^ and outstanding cyclability over 1000 cycles at the current density of 1000 mA g^−1^. Moreover, NPG also shows remarkable electrochemical performance as the anode material for PIBs. A capacity of 194 mAh g^−1^ is obtained at the current density of 1000 mA g^−1^. Even at a high current density of 500 mA g^−1^, NPG exhibits a high reversible capacity along with a stable cycling performance after 500 cycles. In addition, full carbon-based NPG‖LiPF_6_‖AC LICs and NPG‖KPF_6_‖AC PICs show capacities of 98 and 56 mAh g^−1^ at 1 A g^−1^, respectively. Maximum energy densities of 195 and 104.4 Wh kg^−1^ and power densities of 14,983.7 and 14,976 W kg^−1^ can be achieved for the LICs and PICs, respectively, demonstrating the potential applications of NPG anodes. Thus, the arc discharge method is an effective way to prepare graphene doped with various heteroatoms, which can be used as promising electrode materials for high-performance energy storage systems. Moreover, multiple doping represents a smart option to improve the electrochemical performance of electrode materials, including not only graphene, but also other carbon-based materials.

## Electronic supplementary material

Below is the link to the electronic supplementary material.

Supplementary material 1 (PDF 859 kb)
