# Supplementary material for: Nitrogen and Phosphorus Dual-Doped Multilayer Graphene as Universal Anode for Full Carbon-Based Lithium and Potassium Ion Capacitors
Source: Nanomicro Lett. 2019 Apr 3;11:30. doi: 10.1007/s40820-019-0260-6 (PMC7770933; doi:10.1007/s40820-019-0260-6)
Supplement: Supplementary file 1 — Supplementary material 1 (PDF 859 kb) [file 40820_2019_260_MOESM1_ESM.pdf]

Supporting Information for

Nitrogen and Phosphorus Dual-Doped Multilayer Graphene as Universal Anode for Full Carbon-Based Lithium and Potassium Ion Capacitors

Yuting Luan[1], Rong Hu[1], Yongzheng Fang[1], Kai Zhu[1,] *, Kui Cheng[1], Jun Yan[1], Ke Ye[1], Guiling Wang[1], Dianxue Cao[1,] *

[1]Key Laboratory of Superlight Material and Surface Technology of Ministry of Education, College of Material Science and Chemical Engineering, Harbin Engineering University, Harbin 150001, People's Republic of China

*Corresponding authors. E-mail: kzhu@ hrbeu.edu.cn (Kai Zhu); caodianxue@hrbeu.edu.cn (Dianxue Cao)

Supplementary Figures and Tables

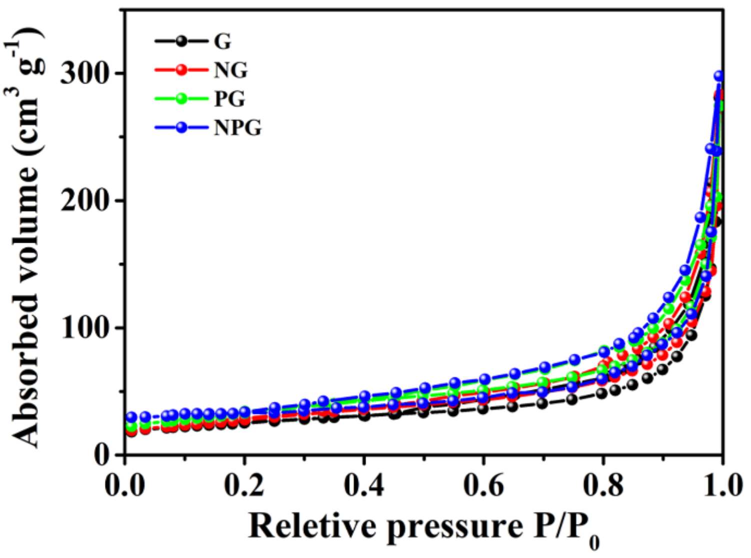

Fig. S1 BET and pore size of G, NG, PG, and NPG

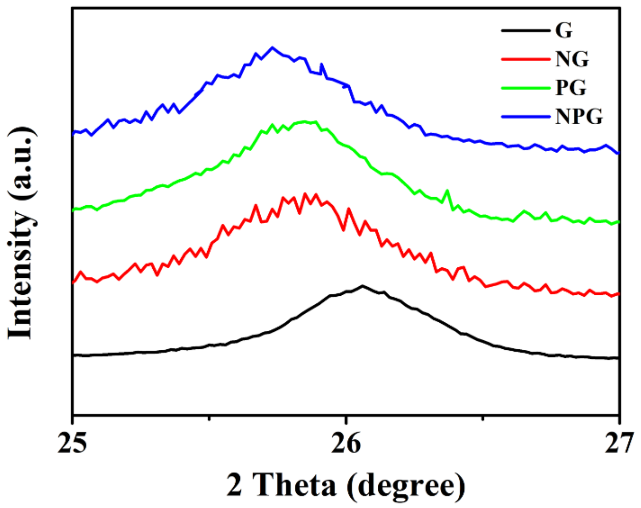

Fig. S2 Partial enlarged drawing of the XRD from the degree from 25 to 27

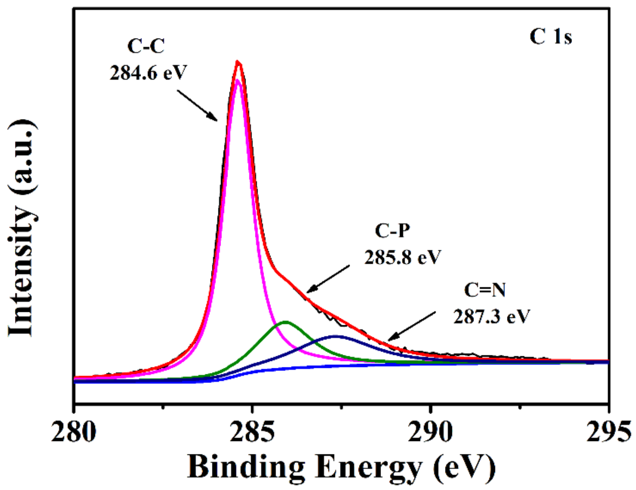

Fig. S3 XPS spectra of C 1s of NPG

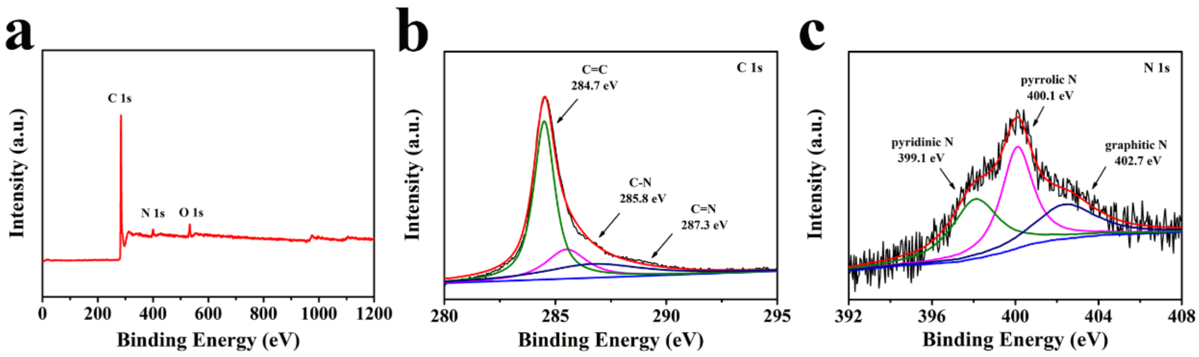

Fig. S4 a Full spectrum and high-resolution XPS spectra of **b** C 1s and **c** N 1s of NG

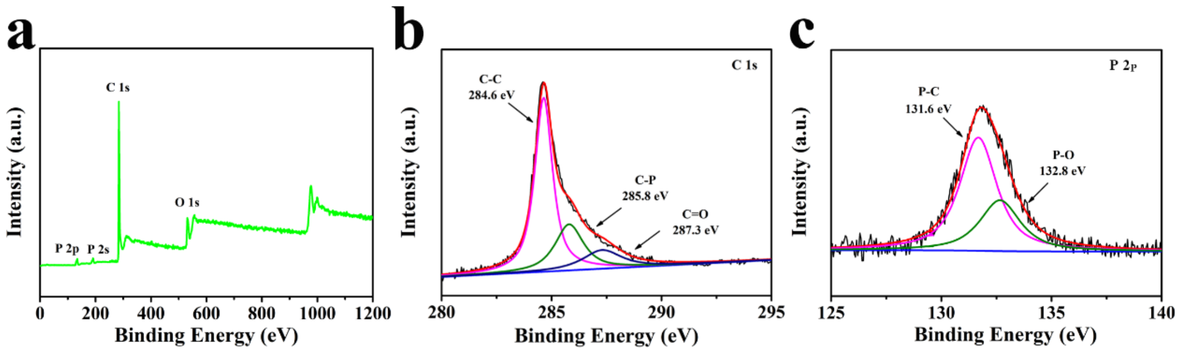

Fig. S5 a Full spectrum and high-resolution XPS spectra of **b** C1s, **c** P 2p and of NPG

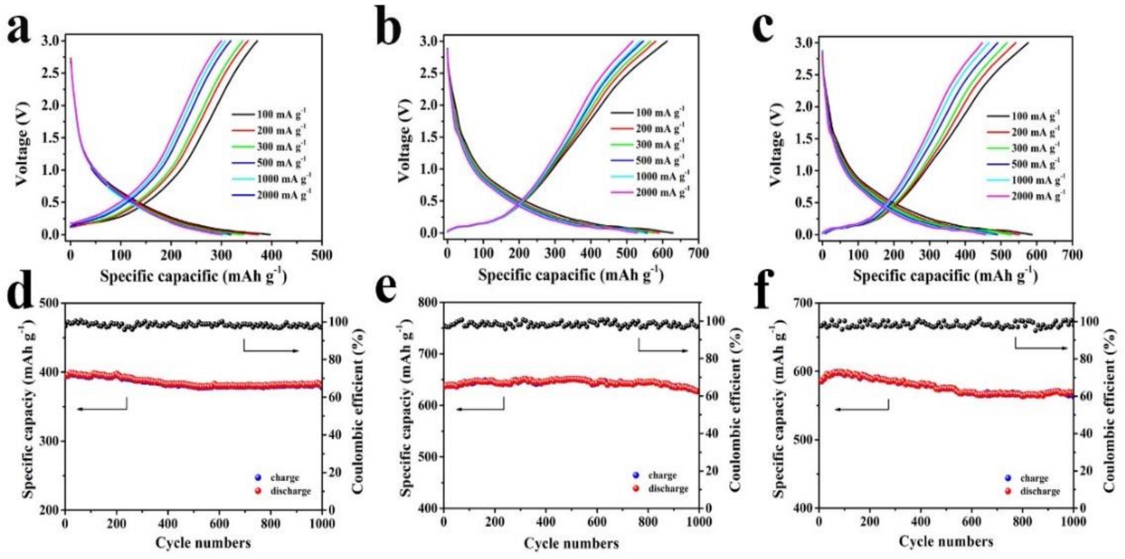

Fig. S6 Typical galvanostatic lithiation/delithiation curves of **a** G, **b** NG, and **c** PG electrodes at different current densities. Cycling stability of **d** G, **e** NG, and **f** PG electrode at the current density of 100 mA g^{-1}

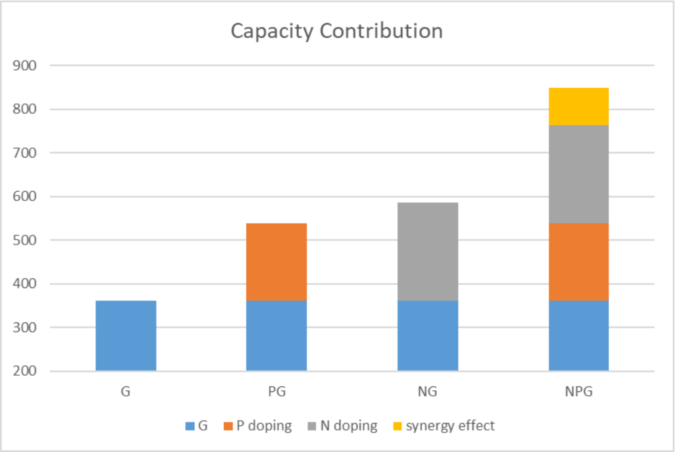

Fig. S7 Qualitative analysis of capacity contribution of G, PG, NG, and NPG at the current density of 0.2 A g

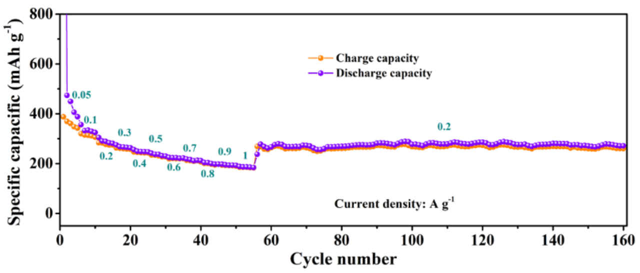

Fig. S8 Rate capabilities of NPG electrodes at different current densities and cycling performance in the potassium battery

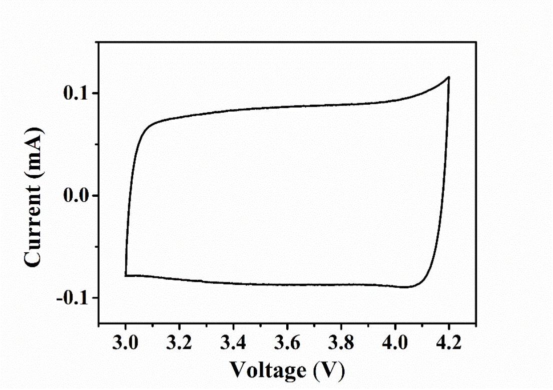

Fig. S9 Electrochemical performance characteristics of AC in a half-cell configuration

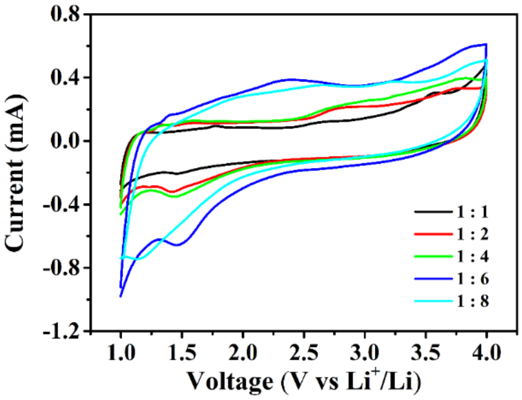

Fig. S10 CV curves of NPG//AC LIC in various weight ratio (Scan rate of 5 mV s^{-1})

Table S1 Nitrogen adsorption isotherms for G, NG, PG, and NPG

Samples	BET (m^2 g^{-1})
**G**	410.9
**NG**	435.7
**PG**	463.7
**NPG**	457.9

Table S2 Calculated R_e and R_{ct} via fitting of the impedance spectra

	R_e (Ω)	R_{ct} (Ω)
Before	2.511	140.7
After	5.461	58.3

Table S3 Comparison of the electrochemical performance of various N-doped carbon, P-doped carbon, and N, P co-doped carbon anodes

Material	Methods	N content	P content	Electrode loading	Current density (mA g^{-1})	Capacity (mAh g^{-1})/cycle	Refs.
N-doped porous graphene	Mix and pyrolysis	12 at%	---	1mg	74.4	900/150	[S1]
N-doped graphene sheets	Mix and pyrolysis	19.5 at%	---	Thickness 20 mm on Cu foils	100	751/108	[S2]
N-doped graphene	Mix and pyrolysis	2.1 at%	---	Disk pieces with diameter of 1.4 cm	50	682/95	[S3]
N-doped graphene sheets	Mix and pyrolysis	13.1 at%	---	0.3-0.5 mg cm^{-2}	100	1050/185	[S4]
P-doped graphene	Mix and pyrolysis	---	1.81 at%	1mg	100	460/80	[S5]
Phosphorus Particles Embedded in Graphene	Mix and pyrolysis	---	35.09 at%	---	500	247/500	[S6]
Graphite/phosphorus	Mixed by grinding	---	28.6 wt%	6 mg cm^{-2}	100	485/50	[S7]
Phosphorus-graphene nanosheet hybrids	Ball-milling	---	70 wt%	0.8-1.5 mg cm^{-2}	260	1570/300	[S8]
Phosphorus/nitrogen-doped graphene paper	Mix and pyrolysis	4.8 wt%	66 wt%	1-3 mg cm^{-2}	1500	809/350	[S9]
P and N dual-doped few-layered porous graphene	Chemical vapor deposition	2.6 at%	0.6 at%	1 mg	1500	750/1000	[S10]
Porous P and N dual doped graphene	Mix and pyrolysis	4.38 at%	1.93 at%	1.5 mg	1675	638/500	[S11]
N and P Dual-Doped Graphene Aerogel	Mix and pyrolysis	4.54 at%	6.72 at%	0.9-1.0 mg cm^{-2}	50	260/50	[S12]
**N-doped graphene sheets**	**Arc-discharge**	**3.31 at%**	**---**	**1 mg cm^{-2}**	**100**	**627/1000**	**This work**
**P-doped graphene sheets**	**Arc-discharge**	**---**	**1.18 at%**	**1 mg cm^{-2}**	**100**	**570/1000**	**This work**
**N, P-co-doped graphene sheets**	**Arc-discharge**	**3.2 at%**	**1.3 at%**	**1 mg cm^{-2}**	**1000**	**787/1000**	**This work**

Supplementary References

[S1]W. Ai, J. Jiang, J. Zhu, Z. Fan, Y. Wang, H. Zhang, Supramolecular polymerization promoted in situ fabrication of nitrogen-doped porous graphene sheets as anode materials for Li-ion batteries. Adv. Energy Mater. **5**(15), 1500559 (2015). https://doi.org/10.1002/aenm.201500559

[S2]L.L. Tian, X.Y. Wei, Q.C. Zhuang, C.H. Jiang, C. Wu, G.Y. Ma, Bottom-up synthesis of nitrogen-doped graphene sheets for ultrafast lithium storage. Nanoscale **6**(11), 6075-6083 (2014). https://doi.org/10.1039/C4NR00454J

[S3]C. He, R. Wang, H. Fu, P.K. Shen, Nitrogen-self-doped graphene as a high capacity anode material for lithium-ion batteries. J. Mater. Chem. A **1**(46), 14586-14591 (2013). https://doi.org/10.1039/c3ta13388e

[S4]L. Tian, S. Li, M. Zhang, S. Li, L. Lin, J. Zheng, Cascading boost effect on the capacity of nitrogen-doped graphene sheets for Li- and Na-ion batteries. ACS Appl. Mater. Interfaces **8**(40), 26722-26729 (2016). https://doi.org/10.1021/acsami.6b07390

[S5]C. Zhang, N. Mahmood, H. Yin, F. Liu, Y. Hou, Synthesis of phosphorus-doped graphene and its multifunctional applications for oxygen reduction reaction and lithium ion batteries. Adv. Mater. **25**(35), 4932-4937 (2013). https://doi.org/10.1002/adma.201301870

[S6]H. Wang, L.F. Wang, L.C. Wang, Z. Xing, X. Wu et al., Phosphorus particles embedded in reduced graphene oxide matrix to enhance capacity and rate capability for capacitive potassium-ion storage. Chem. Eur. J. **24**(52), 13897-13902 (2018). https://doi.org/10.1002/chem.201802753

[S7]A.J. Bai, L. Wang, J.Y. Li, X.M. He, J.X. Wang, J.L. Wang, Composite of graphite/phosphorus as anode for lithium-ion batteries. J. Power Sources **289**, 100-104 (2015). https://doi.org/10.1016/j.jpowsour.2015.04.168

[S8]Z.X. Yu, J.X. Song, M.L. Gordin, R. Yi, D.H. Tang, D.H Wang, Phosphorus-graphene nanosheet hybrids as lithium-ion anode with exceptional high-temperature cycling stability. Adv. Sci. **2**(1-2), 1400020 (2015). https://doi.org/10.1002/advs.201400020

[S9]C. Zhang, X. Wang, Q.F. Liang, X.Z. Liu, Q.H. Weng et al., Amorphous phosphorus/nitrogen-doped graphene paper for ultrastable sodium-ion batteries. Nano Lett. **16**(3), 2054-2060 (2016). https://doi.org/10.1021/acs.nanolett.6b00057

[S10] X.L. Ma, G.Q. Ning, C.L. Qi, C.G. Xu, J.S. Gao, Phosphorus and nitrogen dual-doped few-layered porous graphene: a high-performance anode material for lithium-ion batteries. ACS Appl. Mater. Interfaces **6**(16), 14415-14422 (2014). https://doi.org/10.1021/am503692g

[S11] X.X. Gu, C.J. Tong, C. Lai, J.X. Qiu, X.X. Huang et al., A Porous nitrogen and phosphorous dual doped graphene blocking layer for high performance Li-S Batteries. J. Mater. Chem. A **3**(32), 16670-16678 (2015). https://doi.org/10.1039/C5TA04255K

[S12] Y.P. Wang, Q. Fu, C. Li, H.H. Li, H. Tang, Nitrogen and phosphorus dual-doped graphene aerogel confined monodisperse iron phosphide nanodots as an ultrafast and long-term cycling anode material for sodium-ion batteries. ACS Sustain. Chem. Eng. **6**(11), 15083-15091 (2018). https://doi.org/10.1021/acssuschemeng.8b03561
